# Dependencies among Editing Sites in Serotonin 2C Receptor mRNA

**DOI:** 10.1371/journal.pcbi.1002663

**Published:** 2012-09-06

**Authors:** Liran Carmel, Eugene V. Koonin, Stella Dracheva

**Affiliations:** 1 Department of Genetics, The Alexander Silberman Institute of Life Sciences, Faculty of Science, The Hebrew University of Jerusalem, Edmond J. Safra Campus, Givat Ram, Jerusalem, Israel; 2 National Center for Biotechnology Information, National Library of Medicine, National Institutes of Health, Bethesda, Maryland, United States of America; 3 James J Peters Veterans Affairs Medical Center, Bronx, New York, United States of America; 4 Department of Psychiatry, Mount Sinai School of Medicine, New York, New York, United States of America; Accelrys, United States of America

## Abstract

The serotonin 2C receptor (5-HT_2C_R)–a key regulator of diverse neurological processes–exhibits functional variability derived from editing of its pre-mRNA by site-specific adenosine deamination (A-to-I pre-mRNA editing) in five distinct sites. Here we describe a statistical technique that was developed for analysis of the dependencies among the editing states of the five sites. The statistical significance of the observed correlations was estimated by comparing editing patterns in multiple individuals. For both human and rat 5-HT_2C_R, the editing states of the physically proximal sites A and B were found to be strongly dependent. In contrast, the editing states of sites C and D, which are also physically close, seem not to be directly dependent but instead are linked through the dependencies on sites A and B, respectively. We observed pronounced differences between the editing patterns in humans and rats: in humans site A is the key determinant of the editing state of the other sites, whereas in rats this role belongs to site B. The structure of the dependencies among the editing sites is notably simpler in rats than it is in humans implying more complex regulation of 5-HT_2C_R editing and, by inference, function in the human brain. Thus, exhaustive statistical analysis of the 5-HT_2C_R editing patterns indicates that the editing state of sites A and B is the primary determinant of the editing states of the other three sites, and hence the overall editing pattern. Taken together, these findings allow us to propose a mechanistic model of concerted action of ADAR1 and ADAR2 in 5-HT_2C_R editing. Statistical approach developed here can be applied to other cases of interdependencies among modification sites in RNA and proteins.

## Author Summary

The serotonin receptor 2C is a key regulator of diverse neurological processes that affect feeding behavior, sleep, sexual behavior, anxiety and depression. The function of the receptor itself is regulated via so-called pre-mRNA editing, i.e. site-specific adenosine deamination in five distinct sites. The greater the number of edited sites in the serotonin receptor mRNA, the lower the activity of the receptor it encodes. Here we used the results of extensive massively parallel sequencing from human and rat brains to elucidate the dependencies among the editing states of the five sites. Despite the apparent simplicity of the problem, disambiguation of these dependencies is a difficult task that required development of a new statistical technique. We employed this method to analyse the dependencies among editing in the 5 susceptible sites of the receptor mRNA and found that the proximal, juxtaposed sites A and B are strongly interdependent, and that the editing state of these two sites is a major determinant of the editing states of the other three sites, and hence the overall editing pattern. The statistical approach we developed for the analysis of mRNA editing can be applied to other cases of multiple site modification in RNA and proteins.

## Introduction

The serotonin receptor 2C (5-HT_2C_R) is widely distributed within the central nervous system [1], [2], where it mediates diverse neurological processes that affect feeding behavior, sleep, sexual activity, anxiety and depression [reviewed in [3], [4]]. The 5-HT_2C_R protein belongs to the G-protein-coupled receptor (GPCR) superfamily and potentiates multiple signal transduction pathways via several different G proteins (Gαq/11, Gα12/13 and Gαi) to modulate effector molecules such as phospholipases C, D and A2, as well as the extracellular signal-regulated kinases 1 and 2 [reviewed in [5], [6]].

The 5-HT_2C_R protein exhibits functional variability that is derived from editing of its pre-mRNA by site-specific adenosine deamination (A-to-I pre-mRNA editing) [6]. Editing of 5-HT_2C_R can produce inosine from adenine at up to five closely-spaced (within a 15 nucleotide segment) position that have been named A, B, E (also known as C'), C, and D sites. Because inosine is read as guanosine by the translational machinery, editing can alter codons for three amino acids in the second intracellular loop of the receptor [7], [8], a region involved in coupling with G-proteins [9]. Combinatorial editing at the five positions can generate up to 32 mRNA variants encoding 24 different receptor isoforms (sites A and B as well as sites E and C are situated in the same codons). The extent of editing is inversely correlated with 5-HT_2C_R functional activity such that the more highly edited isoforms are less active than less extensively edited ones [reviewed in [6]]. The unedited Ile156-Asn158-Ile160 (INI) isoform possesses considerable constitutive and agonist-stimulated activity. In contrast, when the 5-HT_2C_R is edited, its coupling to G-proteins and its affinity for serotonin are drastically reduced. Specifically, experiments in heterologous expression systems have shown that, compared to the INI, the fully-edited Val156-Gly158-Val160 (VGV) 5HT_2C_R isoform (which is edited at all five editing sits) has a 40-fold decreased serotonergic capability to stimulate phosphoinositide hydrolysis due to reduced Gq/11-protein coupling efficiency and decreased coupling to other signaling pathways [7], [10]. In addition, cells expressing more highly edited 5HT_2C_R isoforms such as VGV demonstrate considerably reduced (or absent) constitutive activity compared to cells expressing the non-edited INI isoform [10]. This reduction in coupling efficiency and constitutive activity derives from a difference in the ability of edited 5-HT_2C_R isoforms to spontaneously isomerize to the active conformation (R*), a form of the receptor that efficiently interacts with G-proteins in the absence of agonist [11].

A-to-I editing is catalyzed by specific editing enzymes, RNA-specific adenosine deaminases ADAR1 and ADAR2 [reviewed in [12], [13]]. A-to-I editing most frequently occurs in repetitive RNA sequences (e.g., Alu sequences) located within introns and 5′ or 3′ untranslated regions (UTRs). Although the biological significance of non-coding A-to-I RNA editing remains uncertain, the overall editing levels are higher in human compared to primate brains, thus suggesting a possible contribution of editing to the development of higher brain function [14]–[17]. Site-specific edited substrates have been identified in only a few transcripts, including 5-HT_2C_R mRNA, most of which are expressed in the central nervous system (CNS) and encode proteins involved in neurotransmission [6]. In these protein-coding transcripts, several adenosines are targeted within an imperfect RNA fold-back structure. The features that make RNA prone to site-specific editing are not fully understood, but it is thought that internal mismatches and bulges within double-stranded RNA (dsRNA) are important for the specificity of the ADARs [18]–[20].

Although the specificities of ADAR1 and ADAR2 toward different editing sites often overlap, some sites are edited entirely by one enzyme or the other, and the two enzymes display somewhat different preferences for nearest neighbors of the specific editing sites [19], [21]. Experiments on mouse models with null mutations in one or both ADARs suggest that, within 5-HT_2C_R mRNA, the A site is predominantly edited by ADAR1 and the D site is mostly edited by ADAR2 [22]–[24]. The other sites have the potential to be edited by both ADAR1 and ADAR2. In addition, it has been proposed that there is crosstalk between ADAR1 and ADAR2, and therefore the relative expression of the different ADARs might ultimately influence the pattern of editing [reviewed in [6]]. The mechanism underlying the putative crosstalk is unclear, but because the five 5-HT_2C_R editing sites are closely spaced, editing at one site might lead to perturbation of the dsRNA structure that, in turn, would facilitate further editing at other site(s). Indeed, apparent interdependence of editing among the sites has been previously reported for rodent brain [25], [26].

Serotonin signaling, including 5-HT_2C_R, has been implicated in the etiology of behavioral and psychiatric disorders, and 5-HT_2C_R is considered an important target for pharmacologic intervention [4]. Several groups have recently reported an association between 5-HT_2C_R editing and suicide [27]–[31]. Specifically, our studies suggest that in the three major psychiatric diseases (schizophrenia, bipolar disorder, and major depression) that comprise ∼75% of suicides, suicide is associated with enhanced levels of editing (and by inference, with lower activity) of 5-HT_2C_R in the prefrontal cortex independent of the contributions of the underlying disease [30], [31]. The biological mechanisms that contribute to higher 5-HT_2C_R editing (and therefore, hypoactive receptors) in suicide compared to non-suicide psychiatric patients remain unclear. However, it seems likely that because enhanced editing decreases 5-HT_2C_R activity, the resulting reduction in the receptor function might predispose some individuals to suicide by altering 5-HT_2C_R-dependent signal transduction in critical brain regions. Thus, altered editing mechanisms might be linked to liability for suicide, and detailed understanding of these mechanisms could facilitate the development of unique pharmacological strategies that target suicidal behavior.

Alteration of the 5HT_2C_R function via editing has also been reported in response to spinal cord injury (SCI) in rats [32]. Muscle paralysis after SCI is partly caused by a loss of all brainstem-derived neurotransmitters (including serotonin), which normally modulate motoneuron excitability. Murray et al. examined how motoneurons in the spinal cord of the SCI rats compensated for lost brain-derived neurotransmitters to regain excitability and found that changes in 5-HT_2C_R mRNA editing led to increased expression of the 5-HT_2C_R isoforms that are active without serotonin n [32]. Such constitutive receptor activity restored excitability of the motoneurons in the SCI rats in the absence of serotonin, helping motoneurons recover their ability to produce sustained tail muscle contractions. Accordingly, blocking constitutively active 5-HT_2C_R with specific drugs (SB206553 or cyproheptadine) largely eliminated these calcium currents and muscle spasms, providing a new rationale for antispastic drug therapy.

Recently, we applied the Massively Parallel Sequencing (MPS) technology to quantify 5-HT_2C_R editing in the postmortem human brain and the rat spinal cord specimens [31], [33]. The traditional cloning and sequencing approach [7], [34] relies on sampling a limited population of cloned transcripts (∼20–100), thus producing significant sampling errors that can obscure differences between experimental groups. The use of MPS, which analyzes several hundred thousand 5-HT_2C_R transcripts per specimen, not only allowed us to detect all 32 mRNA variants of 5-HT_2C_R in both species, but substantially increased precision and sensitivity in measuring 5-HT_2C_R editing frequencies for all these mRNA variants. Specifically, a comparison between MPS (over 730,000 reads per subject) and the traditional method (46 clones per subject), performed for the same human subjects and the same brain region, has shown that the mean coefficient of variation of the editing frequencies of all variants in the NGS analysis was approximately one-third that of the traditional method [31].

Here we use the MPS data generated in these recent studies on 5-HT_2C_R editing in the human and rat CNS specimens to comprehensively characterize the dependencies among the 5 different editing sites in the 5-HT_2C_R mRNA. The extremely high number of sequenced transcripts combined with the use of a newly developed rigorous statistical procedure allowed us to elucidate the fine structure of these interactions and compare them between the two species as well as among individuals.

## Results

### Massively parallel sequencing data

5-HT_2C_R mRNA editing was measured in the specimens obtained from the human dorsolateral prefrontal cortex and rat spinal cord. The 101 human subjects comprised 45 individuals diagnosed with major depression, and 56 normal controls [31]. The 19 rats comprised 7 controls and 12 rats whose spinal cord was transacted six weeks prior to the data collection [33]. In these rat specimens, the mRNA levels are assumed to be unaffected by the transaction, being collected from a region above it. Overall, the analysed data included 56,690,398 human reads (an average of 561,291 per subject) and 5,659,108 rat reads (an average of 297,848 per rat) (Supplementary Table S1).

Each measurement (mRNA molecule) is represented by a binary vector indicating the editing states of the five sites A, B, E, C, and D. For example, a measurement in which sites A, B, and D are edited but E and C are not is represented by the binary pattern 11001. For a collection of measurements, we denote the *editing pattern* as the vector 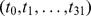, where 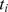 is the number of binary vectors whose decimal representation is 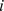.

First, we tested whether the editing patterns of all human normal controls were statistically indistinguishable from the editing patterns of all subjects with major depression. To this end, we conducted a conservative randomization test, whereby the 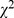-test statistic was repeatedly computed on modified data. In each repetition, we randomly assigned subjects as normal or as depressed, keeping the total number of normal controls and the total number of subjects with major depression fixed. For each repetition, we computed the test statistic of the 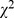-test,
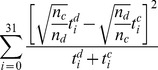
where 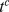 and 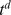 are the editing patterns of normal and depressed samples, respectively, and 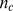 and 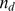 are the total number of measurements from normal controls and from subjects with major depression, respectively. This procedure was repeated 10^6^ times, and the p-value of the test was computed as the number of random test statistics that were larger than the true test statistic. A similar procedure was used to compare normal rats with transacted ones. We found that the editing pattern in normal human controls was indistinguishable from the editing pattern in subjects with major depression (*P* = 0.80), and that the editing pattern in normal rats was indistinguishable from that in transacted rats (*P* = 0.65). This result justifies pooling together all human subjects and all rats for further analysis. Using a similar randomization procedure, we found that the editing pattern in humans is very different from that in rats (*P*<10^−6^) (Supplementary Figure S1).

### Hierarchical clustering of editing sites

Next, we tested for each pair of sites whether their editing patterns were correlated. To this end, we computed the φ-coefficient (which, for binary data, is simply the correlation coefficient; see Methods), and found that all pairs of sites are correlated, either positively or negatively, except for the pair (D,E) in human, and the pair (A,E) in rat (Supplementary Table S2).

**Figure 1:**
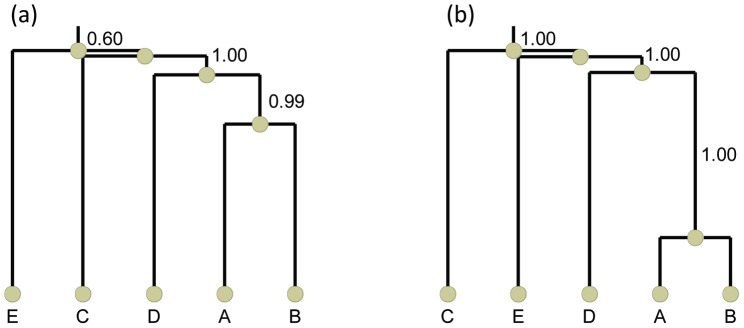
Clustering of the five editing sites using Jaccard distance in (a) human and (b) rat. Each edge is assigned with a confidence level according to the fraction of times by which it was supported by the different individuals.

In order to obtain more detail on the level of dependence between different sites, we followed Ensterö *et al.* [26] and clustered the editing sites (Figure 1). We used the Jaccard distance coupled to single linkage hierarchical clustering (see Methods), but using Dice distance following Ensterö *et al.* [26] had no significant effect on the clustering (Supplementary Figure S2). In order to assign confidence level to the clusters, we repeated the clustering for each individual and measured the fraction of cases in which the cluster was supported (see Methods). In both human and rat the strongest association was observed to exist between sites A and B, to which site D joins next. Sites C and E were more weakly associated with the rest of the editing sites, at least in human, and the order by which they join the dendrogram changed from human to rat.

### Finding the probability model with the best fit to the data

Clustering, by nature, identifies groups of associated sites. However, to obtain finer resolution of the relationship between the sites, we resorted to more elaborate methods. The ultimate description of the dependency between the editing sites would be their joint probability distribution. For five editing sites, there are 8,782 possible joint distribution functions. We enumerated all the 8,782 functions, and ranked them according to how well they fit the data using both maximum-likelihood and Bayesian inference (see Methods).

**Figure 2 pcbi-1002663-g002:**
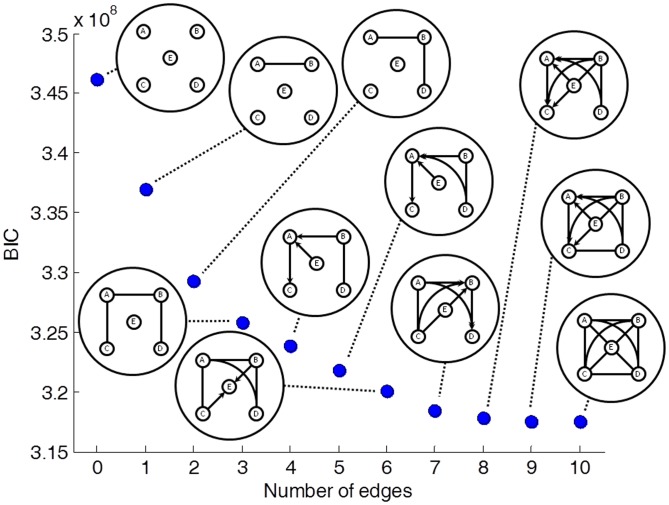
BIC scores (dots) and pDAGs of human models 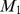 **through** 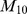 **.** The BIC scores of the models 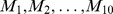 are shown as dots, and the pDAGs of the models themselves are plotted next to each dot. These models represent relationship between sites rather than true causality, as indicated by the fact that some edges reverse their direction in different models. The number of parameters required to describe each of the models is 5 (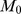), 6 (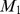), 7 (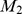), 8 (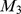), 10 (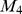), 14 (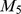), 13 (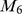), 17 (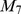), 21 (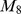), 29 (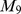), and 31 (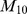).

**Figure 3 pcbi-1002663-g003:**
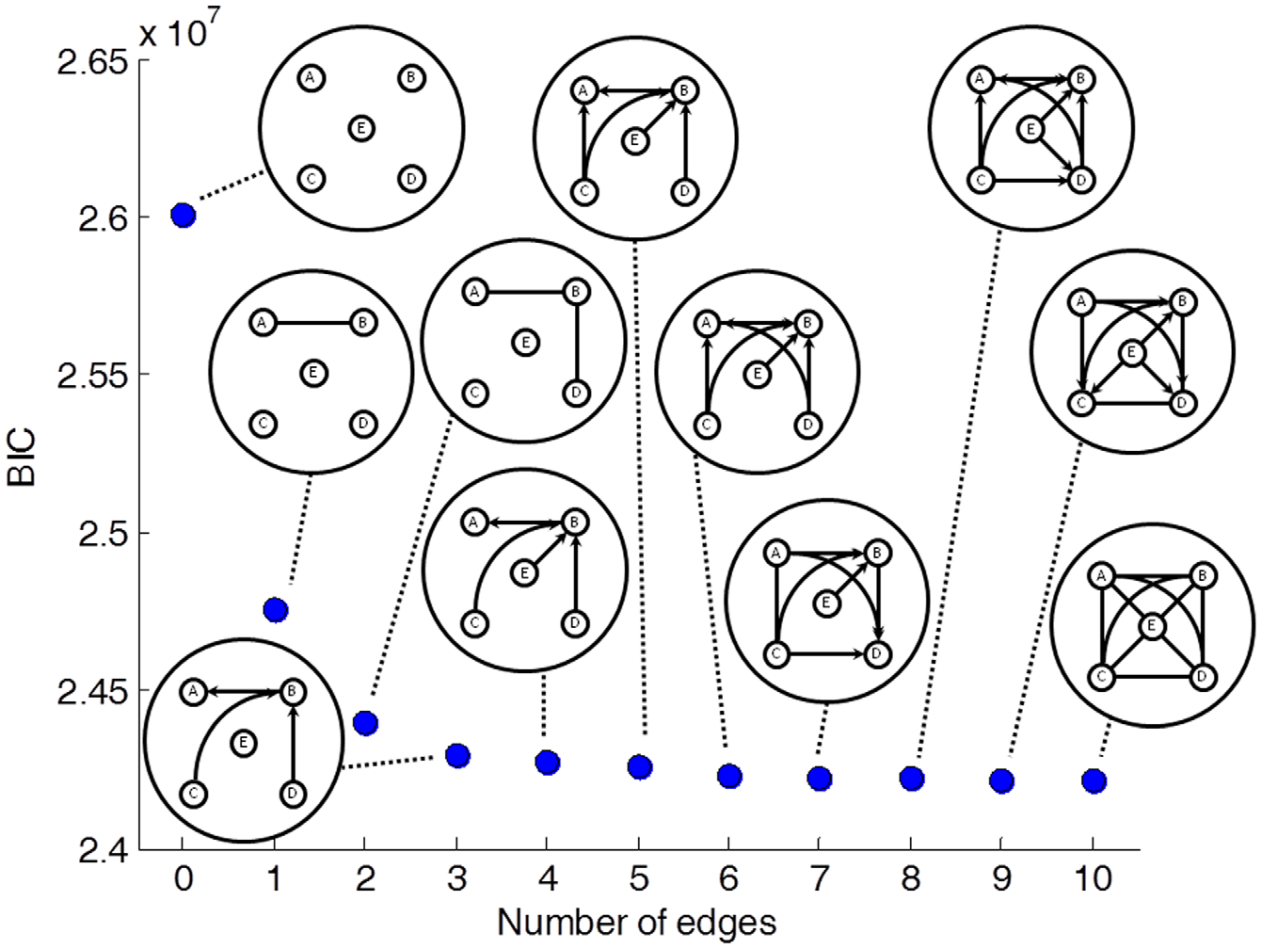
BIC scores (dots) and pDAGs of rat models 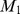 **through** 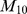 **.** The designations are as in Figure 2. The number of parameters required to describe each of the models is 5 (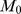), 6 (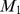), 7 (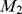), 9 (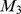), 13 (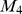), 15 (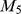), 23 (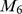), 20 (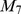), 26 (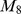), 30 (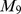), and 31 (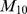).

In both human and rat, and for both maximum-likelihood and Bayesian inference, the best model was the maximally-dependent joint probability distribution, 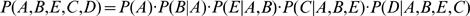. We graphically represent probability models by a pDAG (partial Directed Acyclic Graph), which is a Bayesian network containing a mixture of directed and undirected edges (see Methods). The pDAG of this maximally-dependent model is simply the fully connected undirected graph (Figures 2, 3). This result is consistent with our previous finding that all pairs of sites are significantly dependent.

Such result is expected given the large size of the data. In order to find which edges in the graph are more strongly supported by the data, we divided all the 8,782 probability models into 11 groups according to the number of edges in the corresponding pDAG. The first group consists of all models with zero edges (which is simply the single model 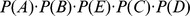), the second group consists of all (ten) models with one edge, etc. Then, we computed the best-fitting probabilistic model within each group. Hereinafter, 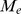 denotes the best-fitting model from within the group of models with 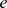 edges. The results for the maximum-likelihood Bayesian Information Criterion (BIC) score (see Methods) in human are shown in Figure 2. Adding an edge to a model always improves its score, 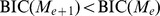, but the improvement becomes smaller as 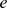 increases. The estimated parameters of each of the best-fitting models are given in Supplementary Table S3.

To further explore the relative impact of the different edges, we ranked the edges by the order in which they first appear in the sequence of models 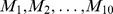. Specifically, the rank of an edge is the smallest integer 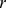 for which 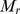 contains this edge (Table 1). Importantly, an edge in 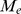 need not necessarily be included in 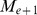, which is the reason why two edges – (B,E) and (E,C) – have the same rank, and why no new edge appeared in 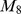. To account for the possibility that edges may disappear or reappear as the number of edges grows, we define for each edge its *support*. The support of an edge with rank 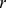 is the fraction of the models 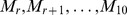 that contain this edge (Table 1). Clearly, the higher the support, the more confident we are that the edge has a unique contribution to making the respective model better fitting the data. The edge (A,B) is the first to appear (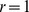), and has a full support (it appears in all the models 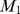 through 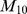), indicating that the dependence between A and B is obviously the strongest among all pairs of sites, in accord with the findings described above. Next appear edges (B,D) and (A,C) that both also have full support. This observation is consistent with the clustering analysis results (Figure 1) but provides more detail on the interdependencies among A, B, and C, D. The next edge to appear is (A,E), but it does not have full support which lowers our confidence in its unique contribution to the score of the best model. The edges that appear in models 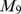 and 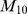 – (C,D) and (E,D) –make (at least qualitatively) only marginal contributions to the score. Repeating the analysis with the maximum-likelihood Akaike Information Criterion (AIC) scores, or with Bayesian scores, gave the same series of best-fitting models 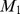 to 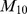 (Supplementary Figure S3).

**Table 1:** Ranking of the strength of dependency of each pair of editing sites in human 5-HT_2C_R mRNA.

rank	Edge	Edge (A,B as F)	Support	Models supporting
1	(A,B)		10/10	1–10
2	(B,D)	(F,D)	9/9	2–10
3	(A,C)	(F,C)	8/8	3–10
4	(A,E)	(F,E)	5/7	4,5,8,9,10
5	(A,D)		6/6	5–10
6	(B,E)		5/5	6–10
	(E,C)	(E,C)	5/5	6–10
7	(B,C)		4/4	7–10
9	(C,D)	(C,D)	2/2	9–10
10	(E,D)	(E,D)	1/1	10

The lower the rank of an edge, and the higher its support, the stronger is the dependency between the pair of editing sites (see text for details). The third column is the same as the second column, except that either A or B are marked as F. The rightmost column lists the models in which the given edge appears.

We conducted the same analysis for the rat data. Here, too, adding edges kept improving the BIC score of the model (Figure 3). The estimated parameters of the best-fitting models are given in Supplementary Table S4. For rat, all edges have full support, which means that an edge with rank 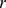 appears in all the models 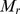 to 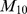 (Table 2). In rat, the two edges with the lowest rank – (A,B) and (B,D) – have the same rank as in human. However, the edge with 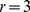 in rat is (B,C) as opposed to (A,C) in the equivalent human 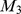 model. Similarly, the edge with 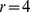 is (A,E) in human, but it is (B,E) in rat. This suggests that the central role of site A in governing the editing state of sites E, C, and D in human is taken by site B in rat. Indeed, referring collectively to site A or site B as F, human and rat show very similar edge rankings (compare Tables 1 and 2). Repeating the analysis with Bayes scores yielded identical series of best-fitting models for rat (Supplementary Figure S4b). Using AIC scores produced only a single difference, in model 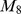. The edge (C,D), which is present in the BIC and Bayes scores, was replaced by the edge (E,C) for the AIC score (Supplementary Figures S4a and S5). However, as we have seen, these edges anyway have marginal contribution to the best-fitting model. On the whole, the information contribution of additional edges dropped much faster for the rat data than it did for the human data (compare Figures 2 and 3), suggestive of a more complex pattern of dependencies among editing sites and accordingly more subtle regulation of the editing process in human brain.

**Table 2:** Ranking of the strength of dependence of each pair of editing sites in rat 5-HT_2C_R mRNA.

Rank	Edge	Edge (A,B as F)	Support	Models supporting
1	(A,B)		10/10	1–10
2	(B,D)	(F,D)	9/9	2–10
3	(B,C)	(F,C)	8/8	3–10
4	(B,E)	(F,E)	7/7	4–10
5	(A,C)		6/6	5–10
6	(A,D)		5/5	6–10
7	(C,D)	(C,D)	4/4	7–10
8	(E,D)	(E,D)	3/3	8–10
9	(E,C)	(E,C)	2/2	9–10
10	(A,E)		1/1	10

The lower the rank of an edge, and the higher its support, the stronger is the dependence between the pair of editing sites (see text for details). The third column is the same as the second column, except that either A or B are marked as F. The rightmost column lists the models in which this edge appears.

The above analysis lacks measure of score variance, thus hindering quantitative evaluation of the significance of each edge to the total score. To overcome this, we repeated the analysis for each individual separately, for both human and rat. In this way, each individual 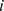 provides its own sequence of best fitting models 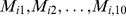, and for each number of edges 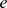 (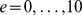), there is now a sequence 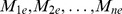 of best models, where 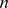 is the total number of individuals. For a certain 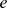, let 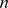 individuals support 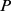 different best-models, 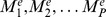, such that 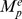 is supported by 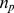 individuals. Let us further assume that we have sorted the sequence 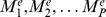 according to the level of support, such that 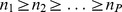. Next, we define a set 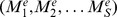 of 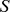 models that are equally supported by the different individuals (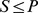). To this end we make a Bonferroni-corrected series of proportion tests, asking whether 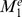 is supported significantly more than the other best-fitting models 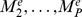. 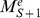 is the first model whose support is significantly lower than that of 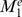. The results for the BIC scores in human at significance level 0.05 are given in Table 3. The results for the AIC and Bayes scores are similar, and are given in Supplementary Tables S5 and S6. As an example, in the BIC score analysis, out of 101 individuals 78 (77.2%) support the single-edge best-fitting model (A→B) (Table 3). The second-supported model 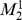 is supported by 21 individuals (20.8%), which is significantly lower than the support for 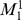 and so in this case 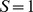 and the set of best fitting models is simply (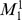). As another example, 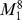 is supported by 14 individuals (13.9%), but this level of support is not statistically different from the support by 3 individuals (3.0%) of the model 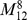, and in this case 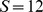. Overall, there is a good agreement between this individual-based analysis and the pooled analysis. The pooled best-fitting model for each 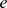 is marked by asterisk in Table 3 and Supplementary Tables S5 and S6, and it is always within the group of models that are equally supported by the different individuals. Very similar results had been obtained for rat (Supplementary Tables S7, S8, S9). Here too, the pooled best-fitting model for each 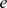 is always within the group of models that are equally supported by the different individuals.

**Table 3:** Statistics on the individual best-models for BIC scores in human.

No. of edges	Model (rank)	Support	Model (edges)
0	(*) 0	101 (100%)	
1	(*) 1	78 (77.2%)	A→B
2	(*) 153	92 (91.1%)	B→A, D→B
3	(*) 833	58 (57.4%)	A→B, A→C, B→D
4	2584	24 (23.8%)	A→B, B→D, E→B, C→A
	(*) 3204	19 (18.8%)	A→C, B→A, E→A, D→B
	8335	19 (18.8%)	A→B, A→C, B→D, C→E
	9752	12 (11.9%)	A→B, A→C, A→D, D→B
5	8342	17 (16.8%)	A→B, B→E, A→C, B→D, C→E
	(*) 3490	15 (14.9%)	A→C, B→A, E→A, D→A, D→B
	9186	10 (9.9%)	A→B, A→C, B→D, E→B, C→E
	9836	10 (9.9%)	A→B, A→C, A→D, C→E, D→B
	2870	9 (8.9%)	A→B, B→D, E→B, C→A, C→B
	2596	8 (7.9%)	A→B, A→D, B→D, E→B, C→A
	180	6 (5.9%)	B→A, E→A, C→A, D→A, D→B
	3210	5 (5.0%)	A→C, E→C, B→A, E→A, D→B
6	(*)10138	21 (20.8%)	A→B, B→E, A→C, A→D, C→E, D→B
	9198	12 (11.9%)	A→B, A→C, A→D, B→D, E→B, C→E
	8356	11 (10.9%)	A→B, A→E, B→E, A→C, B→D, C→E
	2882	10 (9.9%)	A→B, A→D, B→D, E→B, C→A, C→B
	3496	8 (7.9%)	A→C, E→C, B→A, E→A, D→A, D→B
	1986	7 (6.9%)	E→C, B→A, E→A, C→A, D→A, D→B
7	10152	28 (27.7%)	A→B, A→E, B→E, A→C, A→D, C→E, D→B
	(*) 9298	18 (17.8%)	A→B, A→C, A→D, B→D, E→B, C→B, C→E
8	13976	14 (13.9%)	B→E, B→D, B→A, E→A, C→A, C→E, D→A, D→C
	(*) 14620	12 (11.9%)	B→E, A→C, B→C, E→C, B→D, B→A, E→A, D→A
	7012	11 (10.9%)	A→B, A→C, E→C, E→A, E→B, C→B, D→A, D→B
	3511	9 (8.9%)	A→C, B→C, E→C, B→A, E→A, D→A, D→B, D→C
	10159	8 (7.9%)	A→B, A→E, B→E, A→C, A→D, C→E, D→B, D→E
	7108	7 (6.9%)	A→B, A→C, B→C, E→C, E→A, E→B, D→A, D→B
	10300	7 (6.9%)	A→B, A→E, B→E, A→C, A→D, C→B, C→E, D→B
	14448	4 (4.0%)	B→E, B→C, E→C, B→D, B→A, E→A, C→A, D→A
	5812	3 (3.0%)	A→E, B→E, A→D, B→D, B→A, C→A, C→E, D→E
	6959	3 (3.0%)	A→B, E→C, E→B, C→A, C→B, D→A, D→B, D→C
	7036	3 (3.0%)	B→C, E→C, B→A, E→A, E→B, C→A, D→A, D→B
	9208	3 (3.0%)	A→B, A→C, A→D, E→D, E→B, C→B, C→E, D→B
9	(*)14623	31 (30.7%)	B→E, A→C, B→C, E→C, B→D, B→A, E→A, D→A, D→C
	10303	27 (26.7%)	A→B, A→E, B→E, A→C, A→D, C→B, C→E, D→B, D→E
	7111	25 (24.8%)	A→B, A→C, B→C, E→C, E→A, E→B, D→A, D→B, D→C
10	(*) 10655	101 (100%)	A→B, A→E, B→E, A→C, B→C, E→C, A→D, B→D, E→D, C→D

For each family of models with the same number of edges, we report all significantly enriched best-models found among all 101 individuals. The ID of the model is its rank (asterisk marks the best model found in the pooled analysis, see Figure 2). The support is the number of individuals that gave this model as the best-fit model.

**Figure 4 pcbi-1002663-g004:**
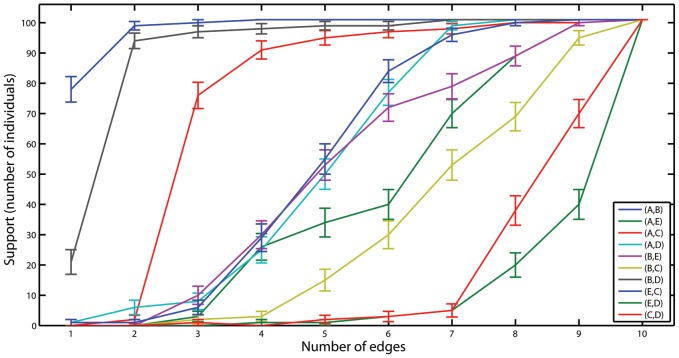
Level of support of each edge in all individual best-fitting models with fixed number of edges. The results are for BIC scores in human.

The individual-based approach can be used not only to re-evaluate the support for the different graphical models but also to perform an edge-by-edge analysis. To this end, we can look at each edge, and count how many times (in either direction) it appears in the sequence 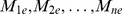. These counts are binomial random variables, so if an edge appears, overall, in the best-fitting model of 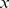 individuals out of a total of 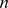 individuals, its variance is 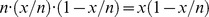. The support of each edge for any 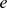 in human is given in Figure 4. Consider, for example, 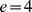. Overall, the 101 individuals support 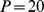 different best-fitting models. Yet, the edge (A,B) appears in all of them, and thus is supported by all the individuals. The edge (B,D) is supported by 98 individuals, or by 97% of the best-fitting models. For each 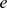, we can take the first 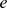 most-supported edges as the *basic set* of edges in the 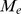 model. Then, we can check how unique is this set of edges by testing (using proportion test) whether the e'*th* supported edge is significantly more supported than the next edges (Table 4). From Table 4 and Figure 4 we see that the first three edges (A,B), (B,D), and (A,C) are clearly more supported than all other edges, in this order. However, the next edges (B,E), (E,C), and (A,D) all have approximately the same support and no one is more significant than the others. The results are almost identical when using AIC or Bayes scores (Supplementary Figures S6 and S7).

**Figure 5:**
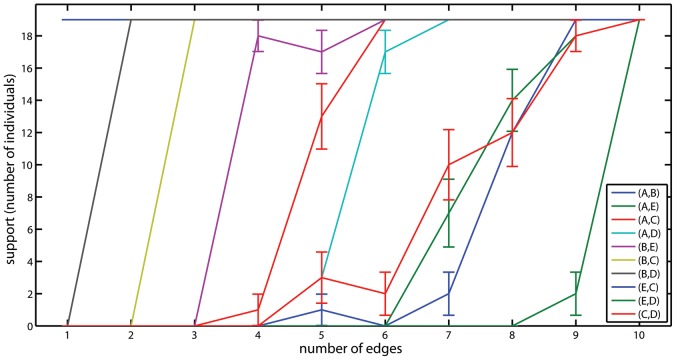
Level of support of each edge in all individual best-fitting models with fixed number of edges. The results are for BIC scores in rat.

Similar analysis for rat shows, in accord with our previous results, a more hierarchical relationship between the edges (Figure 5 and Table 5). Here, the order of importance is clear for the first six edges: (A,B), (B,D), (B,C), (B,E), (A,C), and (A,D). The edges (C,D), (E,D), and (E,C) all have approximately the same support and no one is more significant than the others. The results are almost identical when using AIC or Bayes scores (Supplementary Figures S8 and S9).

**Table 4:** Edges present in the best-fitting models in human (BIC scores).

Number of edges	Number of unique best-fitting models ( 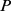 )	Basic set of edges	Additional edges
1	4	(A,B)	
2	5	(A,B), (B,D)	
3	13	(A,B), (B,D), (A,C)	
4	20	(A,B), (B,D), (A,C), (B,E)	(E,C), (A,E), (A,D)
5	22	(A,B), (B,D), (A,C), (B,E), (E,C)	(A,D)
6	25	(A,B), (B,D), (A,C), (B,E), (E,C), (A,D)	
7	20	(A,B), (B,D), (A,C), (B,E), (E,C), (A,D), (A,E)	
8	26	(A,B), (B,D), (A,C), (B,E), (E,C), (A,D), (A,E), (B,C)	
9	15	(A,B), (B,D), (A,C), (B,E), (E,C), (A,D), (A,E), (B,C), (C,D)	
10	1	(A,B), (B,D), (A,C), (B,E), (E,C), (A,D), (A,E), (B,C), (C,D), (E,D)	

For each fixed number of **edges** 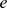 **, we report** the basic set of edges (the most supported 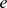 edges), as well as additional edges that are not significantly less supported (at Bonferroni-corrected significance level 0.05).

**Table 5:** Edges present in the best-fitting models in rat (BIC scores).

Number of edges	Number of unique best-fitting models ( 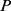 )	Basic set of edges	Additional edges
1	1	(A,B)	
2	1	(A,B), (B,D)	
3	1	(A,B), (B,D), (B,C)	
4	2	(A,B), (B,D), (B,C), (B,E)	
5	6	(A,B), (B,D), (B,C), (B,E), (A,C)	
6	2	(A,B), (B,D), (B,C), (B,E), (A,C), (A,D)	
7	3	(A,B), (B,D), (B,C), (B,E), (A,C), (A,D), (C,D)	(E,D)
8	9	(A,B), (B,D), (B,C), (B,E), (A,C), (A,D), (E,D), (E,C)	(C,D)
9	7	(A,B), (B,D), (B,C), (B,E), (A,C), (A,D), (E,D), (E,C), (C,D)	
10	1	(A,B), (B,D), (B,C), (B,E), (A,C), (A,D), (E,D), (E,C), (C,D), (E,D)	

For each fixed number of edges 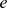, we report the basic set of edges (the most supported 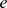 edges), as well as additional edges that are not significantly less supported (at Bonferroni-corrected significance level 0.05).

## Discussion

Here we analysed interdependencies among editing sites within mRNA of 5-HT_2C_R. The studies were performed using available data sets for the human dorsolateral prefrontal cortex and rat spinal cord tissues. Alterations in 5-HT_2C_R editing in these particular species and CNS regions were reported in connection to completed suicide and in response to SCI, respectively [30]–[32]. Thus, detailed understanding of editing mechanisms in these particular areas of the human and mouse CNS are expected to aid in the development of unique pharmacological strategies that target suicidal behavior as well as SCI-related spasticity.

**Figure 6:**
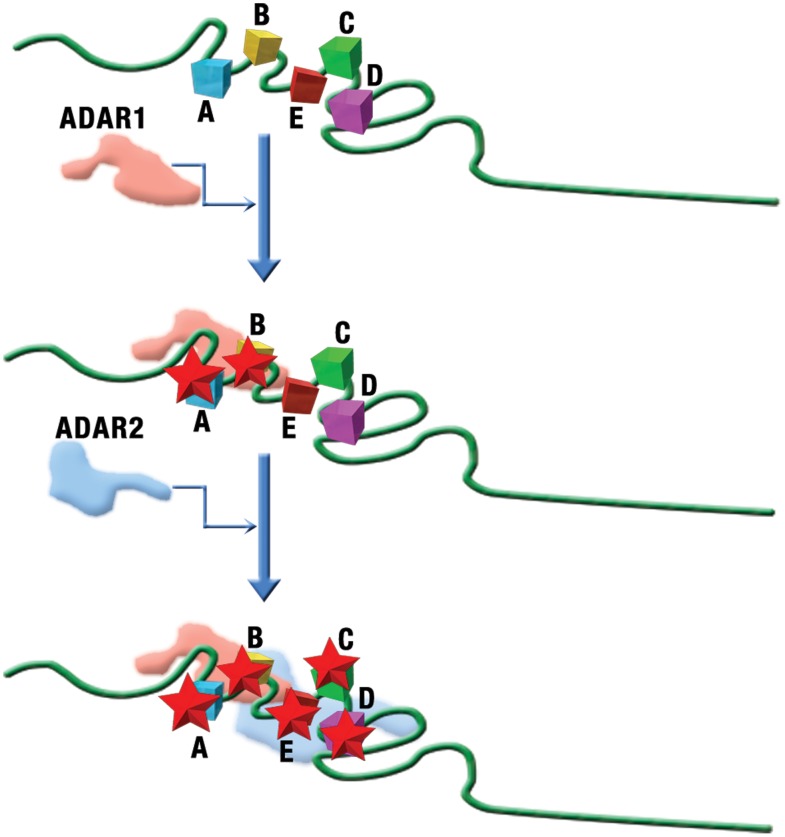
A hypothetical mechanistic model of concerted action of ADAR1 and ADAR2 in 5-HT_2C_R mRNA editing. The squares denote the 5 distinct editing sites and the stars denote editing. The figure is not to scale.

The dependencies among editing sites described here allow us to propose a hypothetical mechanistic model for the concerted action of ADAR1 and ADAR2 in 5-HT_2C_R editing. Given that the dependence between sites A and B was by far the strongest revealed (see Figures 1–5) and that these sites are adjacent in 5-HT_2C_R mRNA, we speculate that ADAR1 that is known to be responsible for editing at A [22] also edits B. Moreover, the strong connection between sites A and B mechanistically might stem from editing of both sites by the same ADAR1 molecule without dissociation of the enzyme from the mRNA (Figure 6). Given that site D, known to be edited by ADAR2 [22], is next after sites A and B in terms of the strength of the dependency, followed by site C, we further speculate that editing of sites A and B by ADAR1 affects the RNA structure such that binding of ADAR2 followed by editing at site D and possibly the two remaining sites is enhanced (Figure 6). A more far reaching implication is that the apparent primary role of ADAR1 in 5-HT_2C_R editing makes it the most attractive target for pharmacological intervention in the associated psychiatric disorders. It is worth noting that such intervention would not interfere with the essential editing of the GluR2 subunit of the AMPA receptor that is primarily dependent on ADAR2 [35].

The results reported here show that for both human and rat 5-HT_2C_R, the editing states of the physically proximal sites A and B are highly dependent. In contrast, the editing states of sites C and D, which are also physically close, seem not to be directly dependent, but rather indirectly linked through the dependencies of C and D on sites A and B, respectively. The results also reveal pronounced differences between the editing patterns in humans and rats: in humans site A has the key role in determining the editing state of the other sites whereas in rats this role belongs to site B. Although not detected by the simple analysis of the dependencies among the editing sites, computing the best-fitting probabilistic models shows that the editing state of site E is strongly dependent on the state of site A in human and on the state of site B in rat (Tables 1 and 2). Furthermore, the structure of the dependences between the editing sites is simpler in rats than it is in human implying more complex regulation of 5-HT_2C_R editing and by inference function in human brain. Mechanistically, the differences between the emerging patterns of editing regulation in humans and rats could be underpinned by the notable differences in the predicted secondary structures of the respective pre-mRNA regions [6].

To conclude, the results of the exhaustive analysis of 5-HT_2C_R editing patterns described here indicate that sites A and B strongly depend on each other in both human and rat, and that the editing state of these two sites is a key determinant of the editing state of the other three sites, and hence the overall editing pattern. The direct dependencies among the editing states of sites E, C, and D are much weaker, and the observed dependencies are probably an indirect effect of the dependency of those three sites on editing in sites A and B. Taken together, these findings allowed us to propose a mechanistic model of concerted action of ADAR1 and ADAR2 in 5-HT_2C_R editing. Methods of statistical inference developed here can be applied to other cases of interdependencies among multiple modification sites in RNA and proteins.

## Methods

The editing state of the five editing sites (A,B,E,C,D) in a single mRNA molecule is represented by a 5-digit binary vector, with one designating an edited site and zero designating an unedited site. The data comprise measurements of the editing state of all five editing sites in 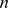 mRNA molecules.

### Statistical test for pairwise correlation between editing sites

We tested whether the editing state of a pair of sites 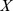 and 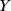 is correlated by computing the contingency table
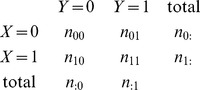
for each individual, where 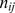 is the number of mRNA molecules in which 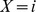 and 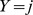 (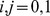) in that individual. Then, the φ-coefficient was computed.
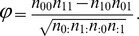
This computation was repeated for all possible pairs in all individuals. Grouping the values from all individuals, the mean and standard deviation were computed for the φ-coefficient for each pair of sites, and the z-test was used to test for significance.

### Clustering of the editing sites

We defined the distance between sites 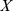 and 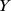 as the Jaccard distance between their binary patterns,
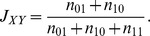
This distance was computed for all pairs of editing sites, and the distance matrix served as input for a single linkage (shortest-distance) hierarchical clustering. Using the Dice distance, as in [26],
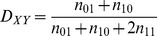
had a negligible effect on the results (Figure 1, Supplementary Figure S2). Edges were given support between 0 and 1 according to the number of individuals in which they are supported.

### Identification of the probabilistic model that best describes the relationship between the editing sites

The editing state of a site is a random variable. Thus, the joint probability distribution 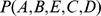 is the ultimate description of the dependencies among the five serotonin receptor editing sites. Any joint probability distribution can be decomposed in many different ways as a product of conditional and marginal probabilities, where each decomposition may represent different dependencies among the sites. For example, the joint probability distribution of two random variables can be decomposed in three ways: 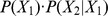, 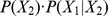, and 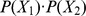. The first two models represent dependency between 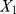 and 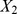, whereas the third model represents independence between 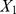 and 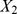. There is a recursive formula for computing the number of possible decomposition for any given number of random variables [36]. In our case, the joint probability distribution of five random variables can be decomposed in 29,281 different ways. Importantly, many of these decompositions are redundant in the sense that several decompositions can describe essentially the same probabilistic model. In the two-random variable example above, Bayes law renders equivalence between the first two decompositions, 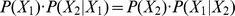. A set of equivalent decompositions is denoted *equivalence class*. There is no known general formula to compute the number of equivalence classes for a given number of random variables. However, there is an algorithm allowing one to tell, given two decompositions, whether they belong to the same equivalence class or not [37].

Here we propose a technique to find the joint probability distribution that fits best to the data. This technique, being exponential with the number of editing sites, is useful when there is a small number of editing sites, as in the present case and in several other functionally important cases of mRNA editing (e.g., kainate 2 glutamate receptor or Ca_V_1.3 channel) [38], [39]. In a nutshell, we scanned through the entire set of 29,281 possible decompositions, and constructed the full set of equivalence classes. Then, we tested which of the equivalence classes fits the data best (see details below).

### Compiling the full set of equivalence classes

In order to enumerate all the possible decompositions of 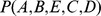, we used Steinsky's ranking algorithm, that allows for a one-to-one mapping between the set of all 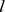 decompositions and the integers 0,1,2,…, 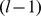 [36]. Then, we scanned through the list of decompositions by a series of pairwise comparisons, and kept only a single decomposition from each equivalence class. In this way, we found that the joint probability distribution of five random variables can be decomposed into 8,782 equivalence classes (Supplementary Table S1).

### Scoring equivalence classes

A Bayesian network provides a compact graphical representation of a decomposition. It is a directed acyclic graph (DAG) in which the nodes are the random variables, and an edge leading from a node to each of its children (a parent of a node 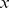 is a node upon which 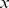 is conditionally dependent in the decomposition). In the context of Bayesian networks, the collection of DAGs that represent equivalence class is called *Markov equivalence class*. For convenience, we shall hereinafter use *probabilistic model* as a synonym to equivalence class or to Markov equivalence class. Bayesian networks have been proved as a very efficient tool to facilitate calculations on probabilistic models.

In order to score how well each probability model fits the observed data, we used two alternative scoring methods. The first is based on a maximum-likelihood (ML) procedure, and the second is based on Bayesian inference. Below, we describe both methods.

#### Maximum-likelihood scoring

Let 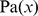 be the set of parents of node 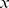. 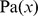 may be empty, or may consists one or more nodes. For example, in the model 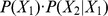, 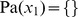, and 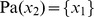. Let 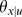 be the probability that 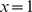 given that the editing state of the parents of 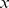 is 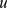. For example, in the model 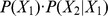, 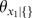 is the probability 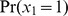, 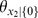 is the probability 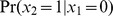, and 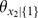 is the probability 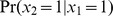. A probabilistic model is characterized by the collection of these parameters, 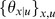. Let 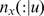 be the number of measurements (mRNA molecules) in which the editing state of 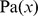 is 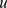. Similarly, let 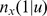 be the number of measurements in which 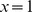 and the editing state of 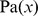 is 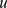. Likewise, 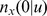 is the number of measurements in which 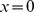 and the editing state of 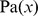 is 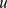. Clearly, 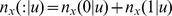. The maximum-likelihood estimate of 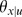 for a given probabilistic model is
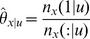

[40], [41]. The log of the maximum likelihood for a given probabilistic model is, up to an additive constant,
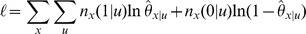
where the first summation is over all the nodes, and the second summation is over all the possible editing states of 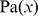. In order to properly compare the different probabilistic models, we have to take into account the fact that the models differ by the number of parameters that characterize them, 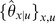. For example, as already pointed out above, the model 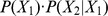 is characterized by three parameters: 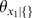, 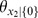, and 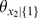. In contrast, the model 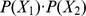 is characterized by only two parameters: 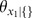, and 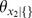. Thus, we employ the Bayesian Information Criterion (BIC) technique [42], and compute for each model the quantity
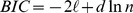
where 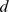 is the number of parameters in the model, and 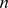 is the total number of measurements (mRNA molecules). The model with the minimum value of BIC is the one that best fits the data.

We also employed a similar method known as the Akaike Information Criterion (AIC) technique [43]. The two techniques yielded the same ranking of the best probability models. In human, BIC and AIC are highly correlated (*ρ* = 0.89), and yield identical order for the first 75 best models. In rat, the correlation between BIC and AIC is somewhat lower (*ρ* = 0.55), but still yield identical order for the first 9 best models.

### Bayesian scoring

The Bayesian learning formalism requires assumptions about the prior probability of the parameters 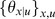. We used the Dirichlet priors, which is the standard choice of priors in this kind of problems because it bears desirable properties such as global and local parameter independence [40]. For each node 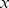, and for each editing state of its parents 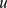, the Dirichlet priors are specified by two parameters that we denote 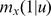 and 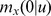. The use of these priors can be conceived as adding another 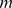 pseudo-measurements to the observed 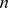 measurements, where 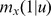 is the number of pseudo-measurements in which 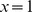 and the editing state of 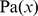 is 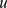, and 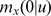 is the number of pseudo-measurements in which 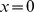 and the editing state of 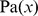 is 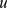. We denote by 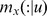 the number of pseudo-measurements in which the editing state of 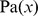 is 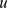, 
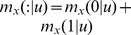
. The Bayesian Score (BS) of a probabilistic model is given by
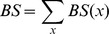
where the summation is over all the nodes, and
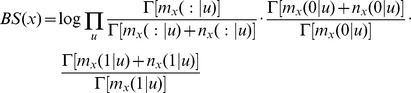
where the product is over all possible editing statees of 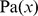, and 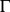 is the gamma function [40].

We generated the set of pseudo-measurements to consist exactly one of each of the possible editing statees of the five editing sites. That is, the pseudo-measurements consist a single measurement 00000, a single measurement 00001, etc. If we denote the number of editing sites by 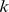 (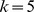), then the set of pseudo-measurements consists of 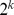 measurements. If we denote the number of parents of node 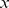 by 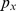, then 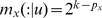, and 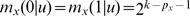. This gives
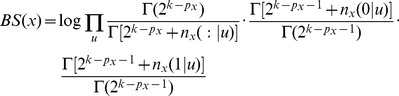
which is just
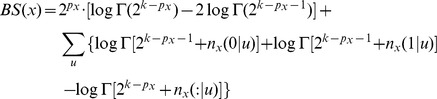
If node 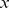 has no parents, then 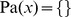, 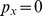, 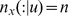, and the formula further simplifies to
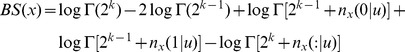



### Visualization

A whole Markov equivalence class can be described by a partial-directed acyclic graph (pDAG) [40], [41], which is a graph made of both directed and undirected edges. If an edge can be oriented differently in DAGs belonging to the same Markov equivalence class, it would be undirected. In this work, whenever a probabilistic model is visualized as a Bayesian network, the pDAG representation is employed.

## Supporting Information

Figure S1 **Frequency of the editing statuses.** **(blue)** human subjects with major depression; **(cyan)** normal human controls; **(orange)** transacted rats; **(red)** normal rat controls.
(DOC)

Figure S2 **Clustering of the five editing sites using Dice distance.** (**a**) human; (**b**) rat.
(DOC)

Figure S3
**a) AIC score, and b) Bayesian score for human models** 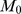 **to** 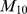 **.** The pDAGs associated with each model are identical to those obtained by the BIC score (Figure 2).(DOC)

Figure S4
**a) AIC score, and b) Bayesian score for rat models** 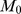 **to** 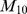 **.** The pDAGs associated with each model are identical to those obtained by the BIC score (Figure 3), except for the 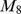 model under the AIC scores, which is depicted in Figure S5.(DOC)

Figure S5
**pDAG for the rat** 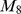 **model.** **a**) as obtained from the BIC and from the Bayes scores; **b**) as obtained from the AIC score. The difference is in the single edge connecting C to D in the BIC and Bayes scores, which is replaced by an edge connecting C and E in the AIC score.(DOC)

Figure S6 **Level of support of each edge in all individual best-fitting models with fixed number of edges.** The results are for AIC scores in human.
(DOC)

Figure S7 **Level of support of each edge in all individual best-fitting models with fixed number of edges.** The results are for Bayes scores in human.
(DOC)

Figure S8 **Level of support of each edge in all individual best-fitting models with fixed number of edges.** The results are for AIC scores in rat.
(DOC)

Figure S9 **Level of support of each edge in all individual best-fitting models with fixed number of edges.** The results are for Bayes scores in rat.
(DOC)

Table S1 **Number and composition of the mRNA molecules collected for human and rat.**
(DOC)

Table S2
**The** 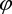 **-coefficient between the editing sites for (a) human, and (b) rat.** All coefficients are significant (FDR corrected), except for the pair (D,E) in human and (A,E) in rat. Errors are standard deviations.(DOC)

Table S3 **The estimated parameters of the best-fitting models in human.** The models are shown in Figure 2.
(XLS)

Table S4 **The estimated parameters of the best-fitting models in rat.** The models are shown in Figure 3.
(XLS)

Table S5 **Statistics on the individual best-models for AIC scores in human.** The statistics for AIC is very similar to BIC, and we report here only those models for which the AIC scores behave differently from the BIC scores (changes are in red). The models are shown in Figure 3.
(DOC)

Table S6 **Statistics on the individual best-models for Bayes scores in human.** The statistics for Bayes score is very similar to BIC, and we report here only those models for which the Bayes scores behave differently from the BIC scores (changes are in red).
(DOC)

Table S7 **Statistics on the individual best-models for BIC scores in rat.** For each family of models with the same number of edges, we report all significantly enriched best-models found among all 19 individuals. The ID of the model is its rank (asterisk marks the best model found in the pooled analysis, see Figure 3). The support is the number of individuals that gave this model as the best-fit model.
(DOC)

Table S8 **Statistics on the individual best-models for AIC scores in rat.** The statistics for AIC is very similar to BIC, and we report here only those models for which the AIC scores behave differently from the BIC scores (changes are in red).
(DOC)

Table S9 **Statistics on the individual best-models for Bayes scores in rat.** The statistics for Bayes score is very similar to BIC, and we report here only those models for which the Bayes scores behave differently from the BIC scores (changes are in red).
(DOC)
