# Supplementary material for: Dependencies among Editing Sites in Serotonin 2C Receptor mRNA
Source: PLoS Comput Biol. 2012 Sep 6;8(9):e1002663. doi: 10.1371/journal.pcbi.1002663 (PMC3435259; doi:10.1371/journal.pcbi.1002663)
Supplement: Figure S3 — a) AIC score, and b) Bayesian score for human models to . The pDAGs associated with each model are identical to those obtained by the BIC score (Figure 2). (DOC) [file pcbi.1002663.s003.doc]

| a) | b) |
| --- | --- |
|  |  |
