# Supplementary material for: Dependencies among Editing Sites in Serotonin 2C Receptor mRNA
Source: PLoS Comput Biol. 2012 Sep 6;8(9):e1002663. doi: 10.1371/journal.pcbi.1002663 (PMC3435259; doi:10.1371/journal.pcbi.1002663)
Supplement: Figure S5 — pDAG for the rat model. a) as obtained from the BIC and from the Bayes scores; b) as obtained from the AIC score. The difference is in the single edge connecting C to D in the BIC and Bayes scores, which is replaced by an edge connecting C and E in the AIC score. (DOC) [file pcbi.1002663.s005.doc]

| a) | b) | |
| --- | --- | --- |
|  |  |  |
