# Supplementary material for: Dependencies among Editing Sites in Serotonin 2C Receptor mRNA
Source: PLoS Comput Biol. 2012 Sep 6;8(9):e1002663. doi: 10.1371/journal.pcbi.1002663 (PMC3435259; doi:10.1371/journal.pcbi.1002663)
Supplement: Table S1 — Number and composition of the mRNA molecules collected for human and rat. (DOC) [file pcbi.1002663.s010.doc]

Table S1: Number and composition of the mRNA molecules collected for human and rat.

| **Pattern [ABECD]** | **Human** | | | **Rat** | | |
| --- | --- | --- | --- | --- | --- | --- |
|  | **Major depression** | **Normal control** | **total** | **Transacted** | **Normal control** | **Total** |
| 00000 | 1718520 | 2924413 | 4642933 | 116161 | 76182 | 192343 |
| 00001 | 714249 | 1069163 | 1783412 | 87975 | 57676 | 145651 |
| 00010 | 648479 | 1151368 | 1799847 | 32022 | 21177 | 53199 |
| 00011 | 445262 | 787179 | 1232441 | 28804 | 20489 | 49293 |
| 00100 | 170220 | 275892 | 446112 | 7094 | 4507 | 11601 |
| 00101 | 62225 | 150645 | 212870 | 5339 | 3188 | 8527 |
| 00110 | 141814 | 189337 | 331151 | 2500 | 1869 | 4369 |
| 00111 | 49123 | 85335 | 134458 | 1347 | 986 | 2333 |
| 01000 | 294091 | 473512 | 767603 | 17571 | 11644 | 29215 |
| 01001 | 320673 | 471193 | 791866 | 25636 | 16641 | 42277 |
| 01010 | 45173 | 66815 | 111988 | 3673 | 2487 | 6160 |
| 01011 | 58399 | 90241 | 148640 | 5445 | 3681 | 9126 |
| 01100 | 7186 | 11983 | 19169 | 473 | 405 | 878 |
| 01101 | 8812 | 17091 | 25903 | 610 | 582 | 1192 |
| 01110 | 3039 | 3225 | 6264 | 76 | 62 | 138 |
| 01111 | 3263 | 4004 | 7267 | 99 | 58 | 157 |
| 10000 | 1284387 | 2087527 | 3371914 | 186397 | 124258 | 310655 |
| 10001 | 1219995 | 2049056 | 3269051 | 102525 | 70898 | 173423 |
| 10010 | 730765 | 1210173 | 1940938 | 40031 | 26681 | 66712 |
| 10011 | 1081623 | 1815858 | 2897481 | 26864 | 17662 | 44526 |
| 10100 | 167280 | 265233 | 432513 | 7755 | 5081 | 12836 |
| 10101 | 175065 | 334430 | 509495 | 4402 | 3418 | 7820 |
| 10110 | 837170 | 1352642 | 2189812 | 10866 | 7311 | 18177 |
| 10111 | 838905 | 1334342 | 2173247 | 4950 | 3862 | 8812 |
| 11000 | 594166 | 970041 | 1564207 | 422962 | 285934 | 708896 |
| 11001 | 2763898 | 4598343 | 7362241 | 1186023 | 818090 | 2004113 |
| 11010 | 994667 | 1482302 | 2476969 | 304155 | 213271 | 517426 |
| 11011 | 4459891 | 7444606 | 11904497 | 610264 | 418174 | 1028438 |
| 11100 | 129707 | 208828 | 338535 | 24610 | 16019 | 40629 |
| 11101 | 338736 | 621390 | 960126 | 51793 | 36340 | 88133 |
| 11110 | 246114 | 427757 | 673871 | 17624 | 11844 | 29468 |
| 11111 | 782574 | 1381003 | 2163577 | 24632 | 17953 | 42585 |
