# Supplementary material for: Dependencies among Editing Sites in Serotonin 2C Receptor mRNA
Source: PLoS Comput Biol. 2012 Sep 6;8(9):e1002663. doi: 10.1371/journal.pcbi.1002663 (PMC3435259; doi:10.1371/journal.pcbi.1002663)
Supplement: Table S2 — The -coefficient between the editing sites for (a) human, and (b) rat. All coefficients are significant (FDR corrected), except for the pair (D,E) in human and (A,E) in rat. Errors are standard deviations. (DOC) [file pcbi.1002663.s011.doc]

Table S2: The
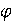
-coefficient between the editing sites for (a) human, and (b) rat. All coefficients are significant (FDR corrected), except for the pair (D,E) in human and (A,E) in rat. Errors are standard deviations.

a)

|  | **B** | **E** | **C** | **D** |
| --- | --- | --- | --- | --- |
| **A** | 0.38±0.04 | 0.12±0.06 | 0.24±0.05 | 0.30±0.04 |
| **B** |  | -0.13±0.05 | 0.13±0.05 | 0.36±0.06 |
| **E** |  |  | 0.18±0.04 | -0.05±0.05 |
| **C** |  |  |  | 0.12±0.04 |

b)

|  | **B** | **E** | **C** | **D** |
| --- | --- | --- | --- | --- |
| **A** | 0.54±0.01 | -0.00±0.01 | 0.08±0.01 | 0.12±0.00 |
| **B** |  | -0.04±0.01 | 0.11±0.01 | 0.26±0.01 |
| **E** |  |  | 0.02±0.01 | -0.04±0.01 |
| **C** |  |  |  | -0.02±0.01 |
