# Supplementary material for: Dependencies among Editing Sites in Serotonin 2C Receptor mRNA
Source: PLoS Comput Biol. 2012 Sep 6;8(9):e1002663. doi: 10.1371/journal.pcbi.1002663 (PMC3435259; doi:10.1371/journal.pcbi.1002663)
Supplement: Table S5 — Statistics on the individual best-models for AIC scores in human. The statistics for AIC is very similar to BIC, and we report here only those models for which the AIC scores behave differently from the BIC scores (changes are in red). The models are shown in Figure 3. (DOC) [file pcbi.1002663.s014.doc]

**Table S5**: Statistics on the individual best-models for AIC scores in human. The statistics for AIC is very similar to BIC, and we report here only those models for which the AIC scores behave differently from the BIC scores (changes are in red).

| **No. of edges** | **Model (rank)** | **Support** | **Model (edges)** |
| --- | --- | --- | --- |
| 6 | 2882 | 9 (8.9%) | A→B, A→D, B→D, E→B, C→A, C→B |
| 7 | 10152 | 27 (26.7%) | A→B, A→E, B→E, A→C, A→D, C→E, D→B |
|  | 3250 | 12 (11.9%) | B→E, B→A, E→A, C→A, C→E, D→A, D→B |
| 8 | 7012 | 12 (11.9%) | A→B, A→C, E→C, E→A, E→B, C→B, D→A, D→B |
|  | 7108 | 6 (5.9%) | A→B, A→C, B→C, E→C, E→A, E→B, D→A, D→B |
|  | 10300 | 6 (5.9%) | A→B, A→E, B→E, A→C, A→D, C→B, C→E, D→B |
| 9 | 10303 | 28 (27.7%) | A→B, A→E, B→E, A→C, A→D, C→B, C→E, D→B, D→E |
|  | 7111 | 26 (25.7%) | A→B, A→C, B→C, E→C, E→A, E→B, D→A, D→B, D→C |
