# Supplementary material for: Dependencies among Editing Sites in Serotonin 2C Receptor mRNA
Source: PLoS Comput Biol. 2012 Sep 6;8(9):e1002663. doi: 10.1371/journal.pcbi.1002663 (PMC3435259; doi:10.1371/journal.pcbi.1002663)
Supplement: Table S7 — Statistics on the individual best-models for BIC scores in rat. For each family of models with the same number of edges, we report all significantly enriched best-models found among all 19 individuals. The ID of the model is its rank (asterisk marks the best model found in the pooled analysis, see Figure 3). The support is the number of individuals that gave this model as the best-fit model. (DOC) [file pcbi.1002663.s016.doc]

**Table S7**: Statistics on the individual best-models for BIC scores in rat. For each family of models with the same number of edges, we report all significantly enriched best-models found among all 19 individuals. The ID of the model is its rank (asterisk marks the best model found in the pooled analysis, see Figure 3). The support is the number of individuals that gave this model as the best-fit model.

| **No. of edges** | **Model (rank)** | **Support** | **Model (edges)** |
| --- | --- | --- | --- |
| 0 | (*) 0 | 19 (100%) |  |
| 1 | (*) 1 | 19 (100%) | A→B |
| 2 | (*) 153 | 19 (100%) | B→A, D→B |
| 3 | (*) 435 | 19 (100%) | B->A, C->B, D->B |
| 4 | (*) 679 | 18 (94.7%) | B->A, E->B, C->B, D->B |
| 5 | (*) 685 | 10 (52.6%) | B->A, E->B, C->A, C->B, D->B |
|  | 682 | 3 (15.8%) | B->A, E->B, C->B, D->A, D->B |
| 6 | (*) 459 | 17 (89.5%) | A->B, E->B, C->A, C->B, D->A, D->B |
| 7 | (*) 2885 | 10 (52.6%) | A->B, A->D, B->D, C->D, E->B, C->A, C->B |
|  | 3415 | 7 (36.8%) | A->B, E->B, C->A, C->B, D->A, D->B, D->E |
| 8 | 7487 | 6 (31.6%) | A->B, E->B, C->A, C->B, C->E, D->A, D->B, D->E |
|  | 2891 | 5 (26.3%) | A->B, A->D, B->D, E->D, C->D, E->B, C->A, C->B |
|  | 4212 | 2 (10.5%) | A->B, A->C, B->C, E->C, A->D, B->D, E->B, D->C |
|  | 3463 | 1 (5.3%) | A->B, A->C, B->C, E->C, E->B, D->A, D->B, D->C |
|  | 4260 | 1 (5.3%) | A->B, A->C, A->D, E->D, E->B, C->B, D->B, D->C |
|  | 4308 | 1 (5.3%) | A->B, A->C, B->C, E->C, A->D, B->D, C->D, E->B |
|  | (*) 6559 | 1 (5.3%) | A->B, E->D, C->D, E->B, C->A, C->B, D->A, D->B |
|  | 9301 | 1 (5.3%) | A->B, A->C, A->D, B->D, C->D, E->B, C->B, C->E |
|  | 18705 | 1 (5.3%) | A->B, E->C, E->D, E->B, C->A, C->B, D->A, D->B |
| 9 | 9707 | 5 (26.3%) | A->B, A->C, B->C, E->C, A->D, E->B, D->B, D->E, D->C |
|  | (*) 4311 | 4 (21.1%) | A->B, A->C, B->C, E->C, A->D, B->D, E->D, C->D, E->B |
|  | 11304 | 4 (21.1%) | A->B, A->C, A->D, C->D, E->B, C->B, C->E, D->B, D->E |
|  | 9307 | 3 (15.8%) | A->B, A->C, A->D, B->D, E->D, C->D, E->B, C->B, C->E |
|  | 7511 | 1 (5.3%) | A->B, A->E, B->E, C->A, C->B, C->E, D->A, D->B, D->E |
|  | 8483 | 1 (5.3%) | A->B, B->E, A->C, B->C, E->C, A->D, B->D, E->D, C->D |
|  | 14623 | 1 (5.3%) | B->E, A->C, B->C, E->C, B->D, B->A, E->A, D->A, D->C |
| 10 | (*) 10655 | 19 (100%) | A→B, A→E, B→E, A→C, B→C, E→C, A→D, B→D, E→D, C→D |
