# Supplementary material for: Dependencies among Editing Sites in Serotonin 2C Receptor mRNA
Source: PLoS Comput Biol. 2012 Sep 6;8(9):e1002663. doi: 10.1371/journal.pcbi.1002663 (PMC3435259; doi:10.1371/journal.pcbi.1002663)
Supplement: Table S9 — Statistics on the individual best-models for Bayes scores in rat. The statistics for Bayes score is very similar to BIC, and we report here only those models for which the Bayes scores behave differently from the BIC scores (changes are in red). (DOC) [file pcbi.1002663.s018.doc]

**Table S9**: Statistics on the individual best-models for Bayes scores in rat. The statistics for Bayes score is very similar to BIC, and we report here only those models for which the Bayes scores behave differently from the BIC scores (changes are in red).

| **No. of edges** | **Model (rank)** | **Support** | **Model (edges)** |
| --- | --- | --- | --- |
| 0 | (*) 0 | 19 (100%) |  |
| 1 | (*) 1 | 19 (100%) | A→B |
| 2 | (*) 153 | 19 (100%) | B→A, D→B |
| 3 | (*) 435 | 19 (100%) | B->A, C->B, D->B |
| 4 | (*) 679 | 18 (94.7%) | B->A, E->B, C->B, D->B |
| 5 | (*) 685 | 10 (52.6%) | B->A, E->B, C->A, C->B, D->B |
|  | 682 | 3 (15.8%) | B->A, E->B, C->B, D->A, D->B |
| 6 | (*) 459 | 19 (100%) | A->B, E->B, C->A, C->B, D->A, D->B |
| 7 | (*) 2885 | 8 (42.1%) | A->B, A->D, B->D, C->D, E->B, C->A, C->B |
|  | 3415 | 8 (42.1%) | A->B, E->B, C->A, C->B, D->A, D->B, D->E |
|  | 2129 | 2 (10.5%) | A->B, E->C, E->B, C->A, C->B, D->A, D->B |
| 8 | 7487 | 6 (31.6%) | A->B, E->B, C->A, C->B, C->E, D->A, D->B, D->E |
|  | 2891 | 5 (26.3%) | A->B, A->D, B->D, E->D, C->D, E->B, C->A, C->B |
|  | 4212 | 3 (15.8%) | A->B, A->C, B->C, E->C, A->D, B->D, E->B, D->C |
|  | 3463 | 1 (5.3%) | A->B, A->C, B->C, E->C, E->B, D->A, D->B, D->C |
|  | 4260 | 1 (5.3%) | A->B, A->C, A->D, E->D, E->B, C->B, D->B, D->C |
|  | (*) 6559 | 1 (5.3%) | A->B, E->D, C->D, E->B, C->A, C->B, D->A, D->B |
|  | 9301 | 1 (5.3%) | A->B, A->C, A->D, B->D, C->D, E->B, C->B, C->E |
|  | 18705 | 1 (5.3%) | A->B, E->C, E->D, E->B, C->A, C->B, D->A, D->B |
| 9 | (*) 4311 | 6 (31.6%) | A->B, A->C, B->C, E->C, A->D, B->D, E->D, C->D, E->B |
|  | 9707 | 4 (21.1%) | A->B, A->C, B->C, E->C, A->D, E->B, D->B, D->E, D->C |
|  | 11304 | 3 (15.8%) | A->B, A->C, A->D, C->D, E->B, C->B, C->E, D->B, D->E |
|  | 9307 | 3 (15.8%) | A->B, A->C, A->D, B->D, E->D, C->D, E->B, C->B, C->E |
|  | 7511 | 1 (5.3%) | A->B, A->E, B->E, C->A, C->B, C->E, D->A, D->B, D->E |
|  | 8483 | 1 (5.3%) | A->B, B->E, A->C, B->C, E->C, A->D, B->D, E->D, C->D |
|  | 14623 | 1 (5.3%) | B->E, A->C, B->C, E->C, B->D, B->A, E->A, D->A, D->C |
| 10 | (*) 10655 | 19 (100%) | A→B, A→E, B→E, A→C, B→C, E→C, A→D, B→D, E→D, C→D |
