# Supplementary material for: Dependencies among Editing Sites in Serotonin 2C Receptor mRNA
Source: PLoS Comput Biol. 2012 Sep 6;8(9):e1002663. doi: 10.1371/journal.pcbi.1002663 (PMC3435259; doi:10.1371/journal.pcbi.1002663)
Supplement: Supplementary file 12 [file pcbi.1002663.s001.doc]

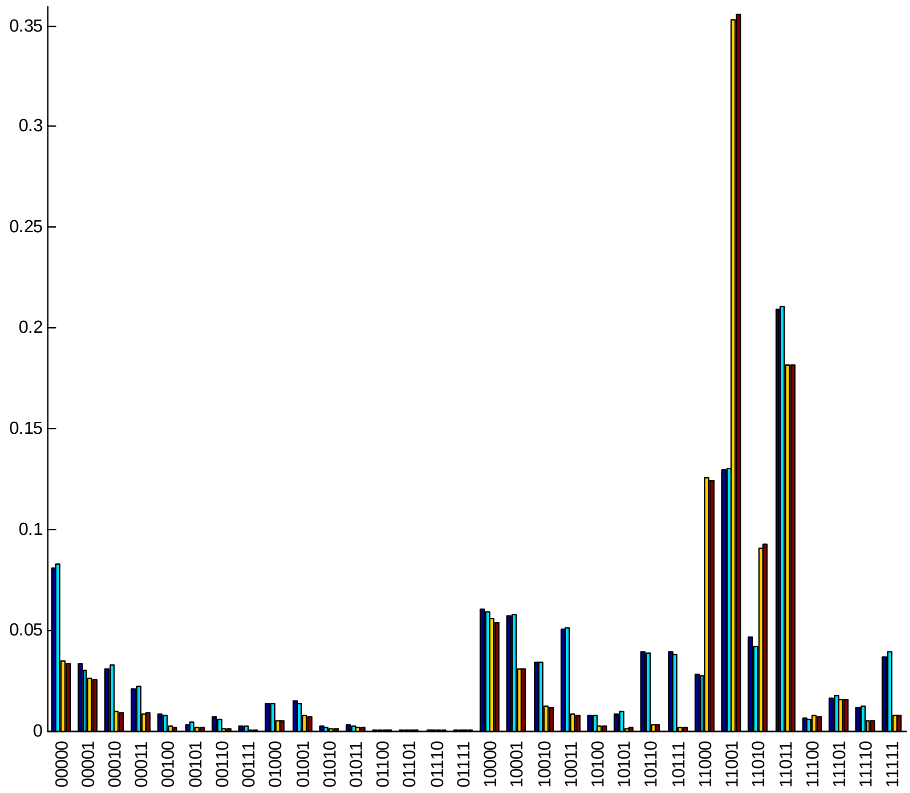
0.35
0.3
0.25
0.2
0.15
0.1
0.05
0
00000
00001
00010
00011
00100
00101
00110
00111
01000
01001
01010
01011
01100
01101
01110
01111
10000
10001
10010
10011
10100
10101
10110
10111
11000
11001
11010
11011
11100
11101
11110
11111
