# Supplementary material for: Dependencies among Editing Sites in Serotonin 2C Receptor mRNA
Source: PLoS Comput Biol. 2012 Sep 6;8(9):e1002663. doi: 10.1371/journal.pcbi.1002663 (PMC3435259; doi:10.1371/journal.pcbi.1002663)
Supplement: Supplementary file 14 [file pcbi.1002663.s007.doc]

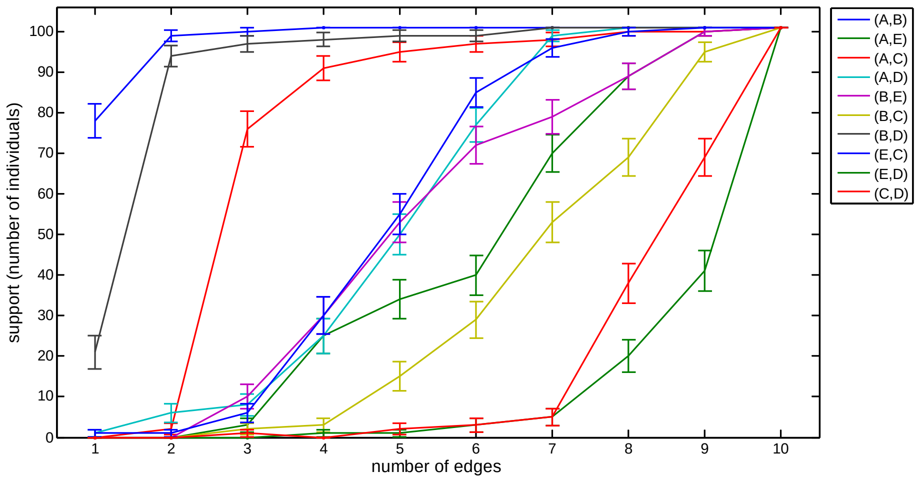

support (number of individuals)
number of edges
(A,B)
(A,E)
(A,C)
(A,D)
(B,E)
(B,C)
(B,D)
(E,C)
(E,D)
(C,D)
